# Reliable *in vitro* studies require appropriate ovarian cancer cell lines

**DOI:** 10.1186/1757-2215-7-60

**Published:** 2014-06-07

**Authors:** Francis Jacob, Sheri Nixdorf, Neville F Hacker, Viola A Heinzelmann-Schwarz

**Affiliations:** 1 Ovarian Cancer Group, Adult Cancer Program, Lowy Cancer Centre, Prince of Wales Clinical School, University of New South Wales, Sydney, Australia; 2 Department of Biomedicine, Gynecological Research Group, University Hospital Basel, University of Basel, Basel, Switzerland; 3 Gynaecological Cancer Centre, Royal Hospital for Women, School of Women’s and Children’s Health, Randwick, Australia

**Keywords:** Epithelial ovarian cancer, Tubal cancer, Peritoneal cancer, Primary cultures, Immortalization

## Abstract

Ovarian cancer is the fifth most common cause of cancer death in women and the leading cause of death from gynaecological malignancies. Of the 75% women diagnosed with locally advanced or disseminated disease, only 30% will survive five years following treatment. This poor prognosis is due to the following reasons: limited understanding of the tumor origin, unclear initiating events and early developmental stages of ovarian cancer, lack of reliable ovarian cancer-specific biomarkers, and drug resistance in advanced cases. In the past, *in vitro* studies using cell line models have been an invaluable tool for basic, discovery-driven cancer research. However, numerous issues including misidentification and cross-contamination of cell lines have hindered research efforts. In this study we examined all ovarian cancer cell lines available from cell banks. Hereby, we identified inconsistencies in the reporting, difficulties in the identification of cell origin or clinical data of the donor patients, restricted ethnic and histological type representation, and a lack of tubal and peritoneal cancer cell lines. We recommend that all cell lines should be distributed *via* official cell banks only with strict guidelines regarding the minimal available information required to improve the quality of ovarian cancer research in future.

## Introduction

Epithelial ovarian cancer (EOC) is the fifth most common cause of cancer death in women and the leading cause of death from gynaecological malignancies [1]. Survival rates have changed little since the early 1980’s despite the use of new chemotherapeutical drugs, with only 40% of all stages and 15-30% of patients with widespread metastatic disease surviving 5 years after the initial treatment [2]. This poor overall prognosis is the result of a combination of factors including a lack of distinctive symptoms and sensitive/specific tumour markers at an early stage, drug resistance for advanced disease, and a limited understanding of the early-initiating events and early stages of EOC development.

### The dualistic paradigm

Among the different tumours arising from the ovary 90% are of epithelial origin [3]. The major histotypes (serous, endometrioid, mucinous, and clear cell) are partly genetically distinguishable as shown by various high-throughput studies in the past fifteen years [4]. Recent findings suggest that epithelial tumours of the ovary may be grouped on the basis of their genetic alterations into a dualistic model that subdivides the various histological types of EOC into two broad categories. The slowly developing tumours (Type I) include low grade serous, endometrioid, mucinous, and a subset of clear cell carcinomas [5-7] and are characterised by genetic alterations in *KRAS*, *BRAF*, *CTNNB1*, *PTEN*, *ARID1A*, *FBXW74*, *PIK3CA*, *PPP2R1A*, and *TGFBR2*[7-12]. The more aggressive Type II tumours harbour mutations in *TP53*, *BRCA1*, and *BRCA2*[8]. A more systematic characterization of Type II tumours, in particular high grade serous ovarian cancers, was performed by The Cancer Genome Atlas (TCGA). The Profiling of 489 samples for differential mRNA and miRNA expression, DNA copy number changes, promoter DNA methylation, and whole exome DNA sequencing revealed that almost all samples comprised *TP53* mutations and significantly recurring somatic mutations in *NF1*, *BRCA1*, *BRCA2*, *RB1*, and *CDK12*[13].

### Ovarian surface epithelium and tubal epithelium as possible tumour origins

The monolayer of epithelial cells covering the outer surface of the ovary (OSE) has traditionally been thought to be the site of origin of epithelial ovarian cancer [1]. This is supported by a recent study focusing on a stem cell niche located at the hilum region and a transitional area between OSE, mesothelium and tubal epithelium. In a comprehensive experimental mouse model the authors demonstrate that stem cell-like OSE cells have the potential to develop into EOC [14]. Another theory proposes the normal epithelium of the fallopian tube (serous), endometrium (endometrioid) and endocervix (mucinous) as the origin of the respective EOC histotypes [15,16]. According to the concept of extra-uterine Müllerian epithelium, the fallopian tube fimbria is proposed to be the primary origin of the high grade serous ovarian carcinoma, the most common EOC subtype and frequently harbouring *TP53* and *IL-6* mutations [17,18]. This is supported by the presence of early neoplastic serous tubal intraepithelial lesions (STIL) in prophylactically removed fallopian tubes of BRCA mutations-carrying women [19-21]. Those tubal fimbria displayed characteristic features such as *TP53* mutations, DNA damage, and secretory cells, suggesting the tubal fimbria as the precursor for high grade serous ovarian cancers [20,22,23]. This was further supported by more recent studies identifying the tubal secretory cells as potential neoplastic precursors at the tubal fimbria. These cells carry *TP53* mutations, show elevated γH2AX expression, a marker of DNA damage, and express Ki-67 and PAX2, two proliferation markers also expressed in serous tubal intraepithelial carcinomas and high grade serous ovarian carcinomas [24-27]. In contrast, epithelial-specific marker such as Calretinin and PAX8 do not seem suitable in the proof of EOC origin [28]. Recently, it has been demonstrated in a *Brca*, *Tp53*, *Pten* genetic mouse model that *de novo* high grade serous ovarian carcinoma are originated from the fallopian tube secretory epithelium and that these tumours are correlated with high grade serous carcinoma tumour markers and genomic alterations of the human TCGA data set [29].

### Serous carcinomas of the ovary, tube and peritoneum

Serous ovarian- (SOC), tubal- (STC), and peritoneal- (SPC) cancers are remarkably similar in term of morphology [30,31], genetics [32], and clinical behaviour and epidemiology [33]. SPC and SOC patients also have a comparable survival rate that, however, is markedly distinct from that of patients with low grade SOC metastasizing to distant locations. Cell lines have long been considered important and useful *in vitro* models to investigate the molecular nature and the pathological processes underlying the development of ovarian, tubal, and peritoneal tumours, and their progression to advanced diseases, and even to search for diagnostic or prognostic tumour markers as well as for therapeutic targets.

### Ovarian cancer cell lines need better characterization

Falling short of the use of *in vivo* animal models, cancer cell lines as *in vitro* models have proven invaluable experimental tools for many decades in basic research. Cancer cell lines can be grown continuously in culture, allowing countless experiments to be performed without the necessary restrictions required for *in vivo* models. However, due to few regulations for the development and testing of these cell lines, the question arises as to the quality of long-time established ovarian cancer cell lines. Often laboratories obtain cell lines from collaborating groups and trust in their identification of cells. Conducting research on the basis of such cell lines means not only a waste of a great deal of money and time but also a risk to steer research in an undesired direction.

It is therefore of great importance to define and establish a world-wide standard applicable to all cell lines that are commercially available for research, in order to ensure that only high-quality cancer cell lines with an unequivocal molecular identity and source are distributed to the research community.

We performed a web search for currently available banks for cells and cell lines using the terms ‘cell bank’, ‘cell lines’, and ‘cell line bank’. Only web pages in English and containing normal or cancer ovarian, tubal, and peritoneal cell lines were included in the study. PubMed (http://www.ncbi.nlm.nih.gov/) was also searched to retrieve references provided by these cell banks reporting additional details of the stocked cell lines. We also included a recent publication in which the copy-number changes, mutations, and mRNA expression profiles in ovarian cancer cell lines were compared to those of high grade SOC (TCGA, http://cancergenome.nih.gov/) [34].

### Commercially available ovarian cancer cell lines

Five cell banks worldwide that stock and distribute normal and/or ovarian cancer cell lines were identified. These are the American Type Culture Collection (ATCC), the European Collection of Cell Cultures (ECACC), the German Collection of Microorganisms and Cell Cultures (DSMZ), the Japanese Collection of Research Bioresources (JCRB), and the National Cell Bank of Iran (NCBI) (Table 1). Remarkably, the Australian cell bank (Cell Bank Australia) does not stock ovarian cell lines.

**Table 1:** Ovarian cancer cell line banks

**ID**	**Name**	**Homepage**
ATCC	American Type Culture Collection	http://www.lgcstandards-atcc.org
ECACC	European Collection of Cell Cultures, a part of the Health Protection Agency	http://www.phe-culturecollections.org.uk/collections/ecacc.aspx
DSMZ	German Collection of Microorganisms and Cell Cultures	http://www.dsmz.de/
JCRB	Japanese Collection of Research Bioresources	http://cellbank.nibio.go.jp/
CellBank Australia	Australian cell bank – Cell Bank Australia	http://www.cellbankaustralia.com/
NCBI	National Cell Bank of Iran	http://ncbi.pasteur.ac.ir/

Our search algorithm retrieved 153 cell lines. ECAAC distributes almost 40% of all publicly available cell lines, followed by JCRB (19%). A number of cell lines (7.2%) are distributed by two or more cell banks. A listing of the ID number, cell line designation (name), origin, and source of the retrieved normal and malignant ovarian, tubal, and peritoneal cell lines is presented in Tables 2 and 3. About two thirds (68.0%) of the normal and ovarian cancer cell lines used in research is of human and about one fourth (23.5%) of Chinese hamster (*Cricetulus griseus*) origin. About 3% originate from mice (*Mus musculus*) and 4.5% from various species such as *Spodoptera frugiperda* (Fall armyworm), *Esox lucius* (Northern pike fish), *Ictalurus punctatus* (Channel catfish), and *Sus domesticus* (Domestic pig). Strikingly, one third of the 104 described human ovarian cancer-derived cell lines were in reality not from ovarian tissue but from peritoneal ascites (21.2%), pleural fluid (3.8%), or metastatic masses (6.7%).

**Table 2:** Human cell lines originated from ovarian cancer or human ovarian surface epithelium

**ID number**	**Cell line**	**Origin**	**Source**
***Homo sapiens *– human**			
1	222		
2	2008	Ovary	
3	2008/C13.R	Ovarian adenocarcinoma	NCBI
4	41M^a^/OAW28	Ovarian cancer ascites	ECACC
5	41 M cisR	Ovarian cancer ascites	
6	59 M	Ovarian cancer ascites	ECACC
7	A2780	Ovarian adenocarcinoma	ECACC
8	A2780ADR	Ovarian adenocarcinoma; A2780	ECACC
9	A2780cis	Ovarian adenocarcinoma; A2780	ECACC
10	A2780 CP	Ovarian adenocarcinoma	NCBI
11	A2780 S	Ovarian adenocarcinoma	NCBI
12	Caov-3	Ovarian adenocarcinoma	ATCC
13	Caov-4	Metastatic fallopian tube mass from ovarian tumour	ATCC/NCBI
14	CH1	Ovarian adenocarcinoma	
15	CH1cisR	Ovarian adenocarcinoma	
16	COLO-704	Metastatic colonic ascites from ovarian tumour	DSMZ
17	COV318	Ovarian cancer ascites	ECACC
18	COV362	Ovarian cancer pleural effusion	ECACC
19	COV362.4	Ovarian cancer pleural effusion; COV362	ECACC
20	COV413A	Metastatic sigmoid mass from ovarian tumour	ECACC
21	COV413B	Metastatic bladder dome mass from ovarian tumour	ECACC
22	COV434	Ovarian granulosa tumour from a solid primary tumour	ECACC
23	COV504	Ovarian pleural effusion	ECACC
24	COV644	Ovarian cancer (primary tumor)	ECACC
25	EFO-21	Ovarian cancer ascites	DSMZ
26	EFO27	Metastatic omental mass from ovarian tumour	DSMZ
27	ES-2	Ovarian adenocarcinoma	ATCC
28	FU-OV-1	Malignant ovarian mass	DSMZ
29	HAC-2	Ovarian cancer cell derived from mesonephros	JCRB
30	Hey-A8	Ovary	CCLE
31	HOSE 6-3	Ovarian surface epithelium	
32	HOSE 17-1	Ovarian surface epithelium	
33	HOSE 105	Ovarian surface epithelium	
34	HOSE 111	Ovarian surface epithelium	
35	HOSE 129	Ovarian surface epithelium	
36	HOSE 130	Ovarian surface epithelium	
37	Hs 38.T	Ovarian teratoma	ATCC
38	Hs 571.T	Ovarian adenocarcinoma	ATCC
39	Hs904.T		
40	IGROV1	Ovarian adenocarcinoma	
41	JHOC-5	Ovarian adenocarcinoma	CCLE
42	JHOM-1	Ovarian adenocarcinoma	CCLE
43	JHOM-2B	Ovarian adenocarcinoma	CCLE
44	JHOS-2	Ovarian adenocarcinoma	CCLE
45	JHOS-4	Ovarian adenocarcinoma	CCLE
46	KURAMOCHI	Ovarian cancer ascites	JCRB
47	MCAS	Ovarian adenocarcinoma	JCRB
48	NCC-OvC-K119	Ovarian adenocarcinoma	JCRB
49	OAW28/41 M	Ovarian cancer ascites	ECACC
50	OAW42	Ovarian cancer ascites	ECACC
51	OC 314	Ovarian cancer ascites	CCLE
52	OC 315	Ovarian adenocarcinoma	CCLE
53	OC 316	Ovarian cancer ascites	CCLE
54	ONCO-DG-1^a^	Ovarian adenocarcinoma	DSMZ
55	OV-7	Ovarian adenocarcinoma derived from solid tumour	ECACC
56	OV17R	Ovarian cancer ascites	ECACC
57	OV56	Ovarian cancer ascites	ECACC
58	OV-58	Ovarian cancer ascites	ECACC
59	OV-90	Ovarian cancer ascites	ATCC
60	OV-1063^a^		
61	OVC1-PI 32	Ovary	NCBI
62	OVCAR-3	Ovarian cancer ascites	ATCC/NCBI
63	OVCAR-4	Ovarian adenocarcinoma	CCLE
64	OVCAR-8	Ovarian adenocarcinoma	CCLE
65	OVISE	Metastatic ovarian adenocarcinoma	JCRB/CCLE
66	OVK18	Ovarian adenocarcinoma	CCLE
67	OVKATE	Ovarian adenocarcinoma	JCRB
68	OVMANA	Ovarian adenocarcinoma	JCRB
69	OVMIU^a^	Ovarian adenocarcinoma	JCRB
70	OVMIU-II^a^	Ovarian adenocarcinoma	JCRB
71	OVSAHO	Ovarian adenocarcinoma	JCRB
72	OVSAYO^a^	Ovarian adenocarcinoma	JCRB
73	OVTOKO	Ovarian adenocarcinoma	JCRB
74	PA-1	Ovarian cancer ascites	ATCC/JCRB/ECACC
75	PA-1/6TG-r	Ovarian cancer ascites	JCRB
76	PEA1	Ovarian cancer pleural effusion	ECACC
77	PEA2	Ovarian cancer ascites	ECACC
78	PEO1	Ovarian cancer ascites	ECACC
79	PEO4	Ovarian cancer pleural effusion	ECACC
80	PEO6	Ovarian cancer ascites	ECACC
81	PEO14^b^	Ovarian cancer ascites	ECACC
82	PEO16	Ovarian cancer ascites	ECACC
83	PEO23^b^	Ovarian cancer ascites	ECACC
84	RKN	Ovarian adenocarcinoma	JCRB
85	RMG-I^a^	Ovarian adenocarcinoma	JCRB
86	RMG-II	Ovarian adenocarcinoma	JCRB
87	RMUG-L^a^	Ovarian adenocarcinoma	JCRB
88	RMUG-S	Ovarian adenocarcinoma	JCRB
89	RTSG^c^	Ovarian adenocarcinoma	JCRB
90	SCC60		
91	SK-OV-3	Ovarian cancer ascites	ATCC/NCBI/ECACC
92	SNU-8	Ovarian adenocarcinoma	CCLE
93	SNU-119	Ovarian adenocarcinoma	CCLE
94	SNU-840	Ovarian adenocarcinoma	CCLE
95	SW 626	Ovarian metastatic mass from colon tumour	ATCC/ECACC
96	TE 84.T	Ovarian adenocarcinoma	ATCC
97	TO14^b^	Metastatic omental mass from ovarian tumour	ECACC
98	TOV-21G	Malignant ovarian mass	ATCC
99	TOV-81D	Malignant ovarian mass	
100	TOV-112D	Malignant ovarian mass	ATCC
101	TYK-nu	Ovarian adenocarcinoma	JCRB
102	TYK-nu.CP-r	Ovarian adenocarcinoma	JCRB
103	UC1-101	Ovarian adenocarcinoma	
104	UC1-107		

^a^Possible cross contamination or misidentification (JCRB, DSMZ: Database of Cross-Contaminated or misidentified cell lines, Capes-Davis, A. and Freshney, R.I. Version 6.7, Table 1 27.6.2011). Cross contaminated with OVCAR-3 (ONCO-DG-1); ^b^All these cell lines were derived from the same patient.

**Table 3:** Non-human cell line originated from the ovary

***Cricetulus griseus *– Chinese hamster**			
105	A2	Ovary	ECACC
106	A2H	Ovary; A2	ECACC
107	AR-EcoScreen	Ovary	JCRB
108	CHO	Ovary	ECACC/NCBI
109	CHO 1–15 500	Ovary	NCBI
110	CHO CD28	Ovary	NCBI
111	CHO-CHRM1	Ovary; CHO-K1	ECACC
112	CHO-CHRM2	Ovary; CHO-K1	ECACC
113	CHO-CHRM5	Ovary; CHO-K1	ECACC
114	CHO DG-44	Ovary	NCBI
115	CHO/dhFr-	Ovary	ECACC/DSMZ/NCBI
116	CHO/dhFr- Ac-free	Ovary; CHO/dhFr-	ECACC
117	CHO-FFAR2	Ovary; CHO-K1	ECACC
118	CHO-GPR120	Ovary; CHO-K1	ECACC
119	CHO/HGPRT	Ovary	JCRB
120	CHO (His9)	Ovary	JCRB
121	CHO-K1	Ovary; CHO	ECACC/JCRB/DSMZ
122	CHO-K1/SF	Ovary; CHO-K1	ECACC
123	CHO-OPRL1	Ovary; CHO-K1	ECACC
124	CHO (pMAM-HSluc)	Ovary	JCRB
125	CHO (pMAM-luc)	Ovary	JCRB
126	CHO Protein-Free	Ovary; CHO	ECACC
127	CHO-SSTR1	Ovary; CHO-K1	ECACC
128	GRL101 (KC7)	Ovary	ECACC
129	GRL101 (MIX)	Ovary	ECACC
130	M1WT3	Ovary; CHO-K1	ECACC
131	NCTC 4206	Peritoneum; B14FAF28-G3	ECACC
132	P22	Ovary	ECACC
133	RR-CHOKI	Ovary; CHO-K1	ECACC
134	T02J-7/10 (CHO-M3 (CHRM3))	Ovary; CHO-K1	ECACC
135	T02J-9/10 (CHO-H2 (HRH2))	Ovary; CHO-K1	ECACC
136	T02J-10/10 (CHO-GCGR (GCGR))	Ovary; CHO-K1	ECACC
137	T26J-1/09 (CHO-Beta-2 (ADRB2))	Ovary; CHO-K1	ECACC
138	T35J-5/09 (CHO-FFAR3 (FFAR3))	Ovary; CHO-K1	ECACC
139	UT-1	Ovary; CHO-K1	ECACC
140	XrS6	Ovary; CHO-K1	ECACC
141	Xrs6-hamKu80	Ovary; CHO-K1	ECACC
***Mus musculus *– mouse**			
142	OV3121	Ovary	JCRB
143	OV3121-ras4	Ovary	JCRB
144	OV3121-ras7	Ovary	JCRB
145	p53-def-MOSE	Ovary	JCRB
146	T-Ag-MOSE	Ovary	JCRB
***Sus domesticus *– Pig**			
147	AVG-16	Ovary follicle	ECACC
***Spodoptera frugiperda *– fall army worm**			
148	Sf 9	Pupal ovary	NCBI/ECACC
149	Sf 9 TitreHigh AC free	Pupal ovary; Sf 9 CL	ECACC
150	Sf 21	Pupal ovary	NCBI/ECACC
151	Sf 21 TitreHigh AC free	Pupal ovary; Sf 21 CL	ECACC
***Esox lucius *– Northern pike fish**			
152	PG	Ovary	ECACC
***Ictalurus punctatus *– channel catfish**			
153	CCO	Ovary	ECACC

It is noteworthy that cell line banks do not stock human cell lines described originating from primary tubal or peritoneal origin. However, only recently the isolation and culturing of normal ovarian and fallopian tube epithelial cells from the same healthy female has been described [35]. This finding may fill the current gap of knowledge and may help clarifying the apparent ambiguity of the origin of ‘ovarian cancer’ and enabling a clear distinction among ovarian, tubal, and peritoneal cancer at their later stages. However, peritoneal cell lines are still not available as are a subset of histologically distinct ovarian cancer cell lines such as borderline cancers, cystadenomas and carcinosarcomas.

The re-naming of cell lines causes constant confusion as respective annotations are often not found in cell banks. For example, 41 M cells are the same as OAW28 cells. Some cell lines have similar names and require caution in the selection of the cell line of choice: a majority of the animal cell lines and several human cell lines are derived from a parental line (e.g. A2780, CHO) and have been modified *in vitro* to display chemo resistance (e.g. cisplatin-resistant A2780CP) or different cellular factors. In addition, the verification of information given by the cell bank is difficult, because not all cell lines are linked to their original publications and their depositors are rarely mentioned.

One apparent shortcoming is that the ethnicity of the ovarian cancer patient from whom the tumour is derived is indicated in only 30.5%. Apart from the JCRB cell bank where all the deposited cell lines were derived from Japanese females (48.3%), the majority of samples where ethnical details are provided were from Caucasian females.

Since we know that different ethnic groups can have a propensity for specific genetic mutations, for example in the *BRCA* and *APC* genes of Ashkenazi Jews [36,37], it is extremely important to have cell lines that represent the spectrum of ethnic groups around the world. This will reduce the risk of an ethnic bias and ensure that research into different ethnic groups will allow the most benefit for these patients.

### The role of genetic changes in the characterization of ovarian cancer cell lines

The (molecular) characterization of EOC in the clinics significantly depends on the presence and type of genetic alterations in the cancer and may define the treatment options and the patients’ outcome. The tumor origin where the cell lines derived from was not precisely provided in 51.2% (Table 4). Considering the clinico-pathological (histotype, FIGO stage, grade) as essential criteria to categorize EOC in type I and II tumours, the respective information provided by cell banks is not sufficient. The data review on available human ovarian cancer cell lines (n = 95) reflects that cell banks provide the histological subtype in 76.8% with discrepancies to original publications (Table 5), stage in 34.7%, and the initial grade in only 20%. In contrast, the information on chemotherapy resistance is provided adequately. Epithelial (-like) cells are characterized with epithelial or stromal markers in more than half (57.9%) of all cell lines, and out of these 85.4% had at least epithelial-like features. Another essential criterion is the doubling time that is provided in only 29.5%.

**Table 4:** Origin of human ovarian cancer cell lines

		**Origin specified (cell line banks)**			
		**Ascites**	**Metastasis**	**Ovary**	**Pleural effusion**
**Origin specified (original references)**	**Ascites**	9	0	5	0
	**Metastasis**	0	2	6	0
	**Not specified**	0	0	11	0
	**Ovary**	0	0	9	0
	**Pleural effusion**	0	0	0	1

**Table 5:** Histotypes of human ovarian cancer cell lines

		**Origin specified (cell line banks)**						
		**Clear cell**	**Endometrioid**	**Mixed**	**Mucinous**	**Other**	**Serous**	**Unknown**
**Origin specified (original references)**	**Clear cell**	6	0	0	0	0	0	0
	**Endometrioid**	0	2	0	0	0	0	0
	**Mixed**	0	0	1	0	0	0	1
	**Mucinous**	0	0	0	3	2	0	0
	**Other**	0	0	0	0	3	0	0
	**Serous**	0	0	0	1	6	7	1
	**Unknown**	0	0	0	0	7	0	1

We also collected and evaluated data provided by cell banks in regards to molecular markers. This information was very limited and only few cell lines were evaluated for expression of progesterone (7.4%) and oestrogen (6.3%) receptors, vimentin (5.3%), *TP53* mutations (4.2%), Her2/neu (3.2%), EpCAM (3.2%), and cytokines 7, 8, 17, 18, and 19 (ranging from 5.3% to 8.4%).

### Potential risks of the use of cell lines for in vitro research

The misidentification and cross-contamination of cell lines is problematic in research and may increase the risk for false results and misinterpretations. The extent of misidentification is documented in a recent study wherein a panel of ovarian and endometrial cell lines was analysed by DNA profiling [38]. The authors found that 8 out of the 51 ovarian cancer cell lines were in fact breast cancer, teratocarcinoma, or cervical cancer cell lines and that2 normal endometrial cancer cells were in fact HeLa cervical cancer or MCF-7 breast cancer cells. Likewise, cross-contamination of cell lines, i.e. the accidental generation of mixed cell cultures, is not a lesser problem. Jäger *et al.* 2013 reported that the popular and frequently used KU7 urothelial carcinoma cell line was cross-contaminated years ago with HeLa cells [39]. Cross-contaminations may occur when multiple cell lines are cultured simultaneously (a practice that should be avoided) and becomes only apparent if multiple morphologies are suddenly observed but fatally remains unnoticed if cells have indistinguishable morphology.

Bacterial/fungal/yeast/mycoplasma contamination presents another problem adversely affecting research results. Of these, mycoplasma species are most likely to be detrimental to cell functioning. Unlike most bacterial, fungal or yeast infections, mycoplasma are macroscopically and microscopically undetectable; it may remain in culture for extended periods of time affecting cell growth, gene expression and overall cell functioning [40]. This may be one reason for why different research groups report contradictory findings. For this reason, Cell Bank Australia has collated a database of known cross-contaminated or misidentified cell lines based on the literature. Other cell banks such as the JCRB have also made an effort to screen the database and identified which of their own cell lines were originally misidentified (Table 1).

The unavailability of a considerable number of *in vitro* cell line models to the research community is also an issue. The problem is two-fold: firstly, there is no quality control of cells generated in individual laboratories when they are not deposited in a professional cell bank. Even when these cells are meticulously generated and cultured, independent quality checks and verifications are not possible. This flaw is overcome by directly contacting the laboratory where the cell lines were generated. This, however, can lead to the second problem; the passing on of cell lines from laboratory to the other, thereby bypassing the critical quality control cell banks. In the past it has been common practice to obtain cells from collaborating groups, and with the required permission, to again distribute these to other laboratories. Whilst this practice is in the spirit of research collaborations, it increases the risk of receiving contaminated or misidentified cell lines that, in turn, can be detrimental to research.

## Conclusions

To ensure a unique quality of cancer research around the world we recommend that all cell lines used for research should be deposited in a cell bank and be readily accessible for all researchers. Ovarian cancer cell bank operators should provide development protocols and comprehensive clinical data for all commercially available cell lines. Depositors of cell lines should ensure that they have carefully collected all relevant clinical information from the donor individuals. This information includes: the exact origin of the cells, the stage during disease progression the cells were taken, the type of therapy the patient underwent prior to sample collection, the data on the patient’s survival, the ethnicity and family history (including known genetic alterations), and the preoperative plasma CA125 levels currently provided by only 5.3% of all human ovarian cancer cell lines. Additionally, we recommend that all cell bank operators conform to the same style of reporting the cell line information and only bank cells where all necessary information is available. This will ensure that the highest standard of research is maintained worldwide. Short tandem repeat (STR) profiling, a highly-sensitive method to detect cellular cross-contamination, should be performed by researchers for all newly generated cell lines and should be confirmed by the cell bank once deposited and prior to the sale of the cells. The service for STR profiling is provided by various laboratories, e.g. American Type Cell culture Collection (ATCC-USA, http://www.atcc.org), China Center for Type Culture Collection (CCTCC, http://www.cctcc.org), Australian Cell Bank ( http://www.cellbankaustralia.com), European Culture Collection of Cell Cultures (ECACC, http://www.hpacultures.org.uk), or German Cell Culture Collection (DSMZ, http://www.dsmz.de). From a recent study that histotyped standard ovarian cancer cell lines by short tandem repeats, immunohistochemistry, and mutation analysis it was concluded that the knowledge of the mutation status of cancer genes such as *ARID1A* and *TP53* and of the general immunoprofile would be beneficial for the determination of the histotype of ovarian cancer cells [41]. Following the model of the Cancer Cell Line Encyclopedia (CCLE), we suggest the establishment of a centralized cell line database that would harbour all the relevant details of new cell lines and would be updated with new details in real time as experimental results are reported in the literature. This is believed to reduce the overlap of research performed and to continually improve the quality and appropriateness of future cell line studies. A cell bank professional with expertise in cancer research would be beneficial for researchers who need advice in correctly choosing the cell line appropriate for a specific research question. The expansion of the current offer of cell lines deposited in the cell banks by additional types of cells is desirable. These include primary, recurrent and metastatic ovarian-, tubal- and peritoneal cancers, a set of cell lines representing all known EOC histotypes, age-matched normal control OSE and tubal cells, and cell lines derived from primary, recurrent and metastatic tumours from the same patients at different progression time points. It is clear that worldwide collaborative efforts are to be taken to reach these recommendations, but we believe that this will be of benefit for the research results in the future.

## Competing interests

The authors declare that they have no competing interests.

## Authors’ contributions

FJ carried out literature research, data analysis and drafted the manuscript. SN carried out literature research drafted the manuscript. NFH drafted the manuscript. VAS conceived the review and drafted the manuscript. All authors read and approved the final manuscript.
